# A comparison of high-throughput SARS-CoV-2 sequencing methods from nasopharyngeal samples

**DOI:** 10.1038/s41598-022-16549-w

**Published:** 2022-07-22

**Authors:** Zuzana Gerber, Christian Daviaud, Damien Delafoy, Florian Sandron, Enagnon Kazali Alidjinou, Jonathan Mercier, Sylvain Gerber, Vincent Meyer, Anne Boland, Laurence Bocket, Robert Olaso, Jean-François Deleuze

**Affiliations:** 1 CEA, Centre National de Recherche en Génomique Humaine, Université Paris-Saclay, 91057 Evry, France; 2 Laboratoire de Virologie ULR 3610, CHU Lille, University of Lille, 59000 Lille, France; 3 Institut de Systématique, Evolution, Biodiversité (ISYEB), Muséum National d’Histoire Naturelle, CNRS, SU, EPHE, UA, CP39, 57 Rue Cuvier, 75005 Paris, France; 4 LabEx GENMED (Medical Genomics), Paris, France; 5 Emergen Consortium, Paris, France

**Keywords:** Next-generation sequencing, Viral genetics, Infectious diseases, Public health, Bioinformatics, Genomic analysis

## Abstract

The COVID-19 pandemic caused by the new Severe Acute Respiratory Syndrome Coronavirus 2 (SARS-CoV-2) continues to threaten public health and burden healthcare systems worldwide. Whole SARS-CoV-2 genome sequencing has become essential for epidemiological monitoring and identification of new variants, which could represent a risk of increased transmissibility, virulence, or resistance to vaccines or treatment. Different next-generation sequencing approaches are used in SARS-CoV-2 sequencing, although with different ability to provide whole genome coverage without gaps and to reliably detect new variants. In this study, we compared the performance of three target enrichment methods (two multiplex amplification methods and one hybridization capture) using nasopharyngeal swabs from infected individuals. We applied these target enrichment methods to the same set of nasopharyngeal samples (N = 93) in high-throughput mode. SARS-CoV-2 genome was obtained using short-read next-generation sequencing. We observed that each method has some advantages, such as high mapping rate (CleanPlex and COVIDSeq) or absence of systematic variant calling error (SureSelect) as well as their limitations such as suboptimal uniformity of coverage (CleanPlex), high cost (SureSelect) or supply shortages (COVIDSeq). Nevertheless, each of the three target enrichment kits tested in this study yielded acceptable results of whole SARS-CoV-2 genome sequencing and either of them can therefore be used in prospective programs of genomic surveillance of SARS-CoV-2. Genomic surveillance will be crucial to overcoming the ongoing pandemic of COVID-19, despite its successive waves and continually emerging variants.

## Introduction

Two years after its emergence, the COVID-19 pandemic caused by the new Severe Acute Respiratory Syndrome Coronavirus 2 (SARS-CoV-2)^1–3^ continues to threaten public health and burden economical and healthcare systems worldwide. While real-time polymerase chain reaction (RT-PCR) is the primary method to diagnose COVID-19 disease^4,5^, it provides no information on viral sequence. However, sequencing is essential for epidemiological monitoring. Firstly, it allows to monitor the adaptive evolution of the virus, such as the emergence of new variants^6–11^. Secondly, it allows to trace the route of transmission and to detect new local clusters^12–15^.

Over 259 million confirmed COVID-19 cases since the onset of the pandemic^16^ have provided ample opportunity for new mutations to occur in the SARS-CoV-2 genome, resulting in new viral strains, some of them classified by the WHO as variants of concern (VOC) for their increased transmissibility, virulence, or resistance to vaccines or treatment. The five variants classified as VOC at the time of writing are: firstly, variant Alpha (B.1.1.7) first detected in the United Kindom, with increased transmissibility^17^ and virulence^18^. Secondly, variant Beta (B.1.351) first detected in South Africa, with likely increased transmissibility or immune escape^19^. Thirdly, variant Gamma (P.1) first detected in Brazil, with increased transmissibility and virulence^20^. Fourthly, variant Delta (B.1.617.2) first detected in India, with increased transmissibility^21^ and mildly decreased vaccine effectiveness^22^, which is the dominant variant today. And most recently, variant Omicron (B.1.1.529) first detected in South Africa, its transmissibility and virulence properties still to be determined^11^.

As worldwide vaccination programs advance, the selective pressure on SARS-CoV-2 evolution towards vaccine-resistant strains is increasing and it may be only a matter of time before the emergence of a new, vaccine resistant strain. Sequencing of vaccine breakthrough infection cases becomes necessary, because early detection and containment of any vaccine resistant strains is of utmost importance for the successful management of the pandemics in the future^23–25^. Early detection of recombinant strains with possibly new properties^26–28^ is equally important. The continued emergence of new VOC and particularly the risk of developing vaccine resistance call for large-scale genomic surveillance of SARS-CoV-2 worldwide^9,29,30^.

Different next-generation sequencing approaches have been used in SARS-CoV-2 sequencing, each with a different ability to provide representative whole genome coverage without gaps and to reliably detect new variants. Several studies have compared shotgun metagenomics, target enrichment by multiplex amplification, target enrichment by hybridization capture, as well as long-read single molecule sequencing^13,31–35^. However, most published benchmarking studies are based on a small number of patient samples, or on RNA extracted from viral culture spiked into human RNA, or on synthetic viral RNA, and are therefore inconclusive for large-scale epidemiological monitoring studies from real patient samples of nasopharyngeal swabs. In this study, we compare the performance of three target enrichment methods applied to the same patient samples of nasopharyngeal swabs (N = 93) in high-throughput mode and we discuss their potential for large-scale sequencing projects.

## Results

Clinical samples of nasopharyngeal swabs were sequenced in this study in order to evaluate three different methods of SARS-CoV-2 genome target enrichment. Aliquots of the same samples were subjected to target enrichment by two amplicon-based approaches (N = 93): *CleanPlex* SARS-CoV-2 Panel (Paragon Genomics) and Illumina *COVIDSeq* Test (Illumina Inc), and a hybridization capture approach (N = 85): *SureSelect* XT HS2 RNA System (Agilent Technologies).

Samples tested positive for SARS-CoV-2 by RT-PCR with uniform low C_T_ values (C_T_ < 20) were selected for this study to focus on the performance of each method in ideal conditions, thus avoiding stochastic effects such as PCR bias or sporadic contamination that are common when working with limited viral material. In order to compare data generated with equivalent sequencing effort for each method, we downsampled the raw data to 2 M read pairs per library. We examined the mapping rate, depth, breadth and uniformity of coverage; compared the resulting variant profiles; and generally evaluated the pros and cons of each method.

### SARS-CoV-2 mapping rate

**Figure 1:**
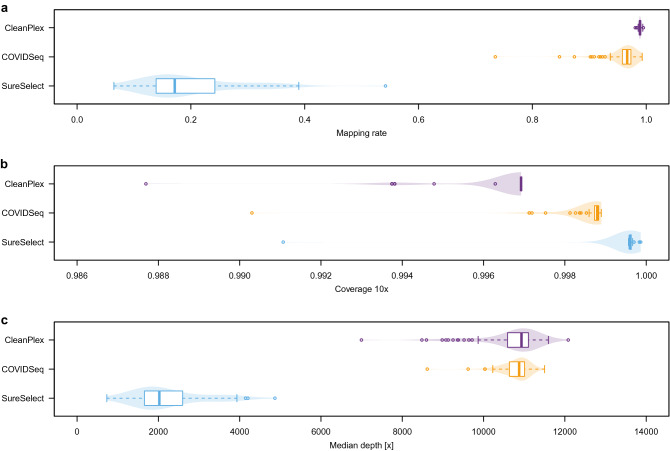
Comparison of target enrichment kits performance. (**a**) SARS-CoV-2-specific mapping rate. (**b**) Breadth of coverage of the SARS-CoV-2 genome, where the depth of coverage is at least 10× . (**c**) Median depth of coverage.

The target is different for each enrichment method benchmarked in this study: CleanPlex targets SARS-CoV-2 genome, COVIDSeq targets SARS-CoV-2 genome but also several human mRNA loci for internal control, and SureSelect targets all human coronaviruses including SARS-CoV-2 (see “Materials and methods” for details). Consequently, the specificity for SARS-CoV-2 varies among kits by design. To assess mapping rate, we used non-parametric Friedman test after having rejected the assumption of normality (Shapiro–Wilk test, p < 0.01 for all three kits). The mapping rate differed significantly among kits (Friedman test, p < 0.01) as displayed in Fig. 1a.

Both amplicon-based approaches had an excellent mapping rate, with an average 98.9% for CleanPlex and 95.8% for COVIDSeq. The mapping rate of COVIDSeq is close yet significantly lower than CleanPlex (Nemenyi post hoc test, p < 0.01). With an average mapping rate of 19.9% to SARS-CoV-2 genome, SureSelect was significantly lower than either of the amplicon methods (Nemenyi post hoc test, both p < 0.01). The mapping rates reflect the respective kit designs: among the reads not mapping to SARS-CoV-2, the proportion of reads that align to human genome is 10% for SureSelect, 3% for COVIDSeq, and 0% for CleanPlex.

### Breadth of coverage

We examined the breadth of coverage to identify possible gaps or areas of low depth of coverage, which would affect variant calling in the concerned region. All three methods achieved above 99% breadth of coverage at 10× as shown in Fig. 1b. The differences were small yet significant (Friedman test, p < 0.01). SureSelect gave systematically the best results (on average 99.95%) while both amplicon-based approaches were slightly lower (COVIDSeq 99.86% and CleanPlex 99.65%); all three pairwise comparisons were significant (Nemenyi post hoc test, all p < 0.01). The difference is likely due to the design of amplicon primer panel, which does not cover several dozens of nucleotides (nt) at the beginning and end of the viral genome for both amplicon methods.

### Depth of coverage

**Figure 2:**
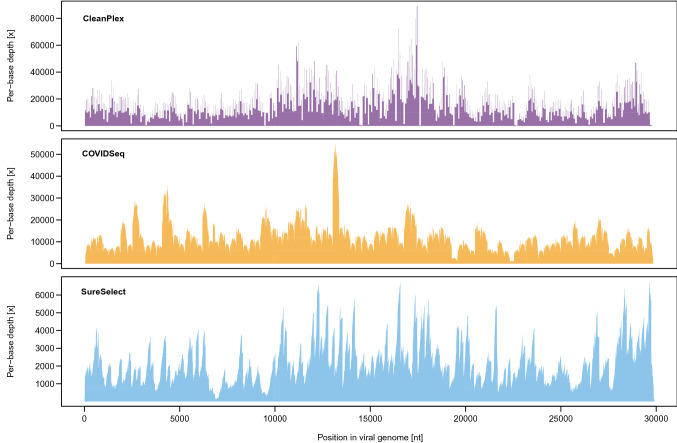
Depth of coverage profile of a typical library constructed with different kits.

Next, we examined the depth of coverage for each method. The median depth of coverage differed significantly among the three methods (Friedman test, p < 0.01). SureSelect yielded significantly lower median depth of coverage (on average 2234 ×) than both COVIDSeq (10,785 ×) and CleanPlex (10,679 ×) as shown in Fig. 1c (Nemenyi post hoc test, both p < 0.01); the latter two were not significantly different (Nemenyi post hoc test, p = 0.607). The lower depth of coverage for SureSelect is likely due to the lower mapping rate as mentioned above. An example of depth of coverage profile of a typical library prepared by the three methods is shown in Fig. 2.

### Coverage uniformity

**Figure 3:**
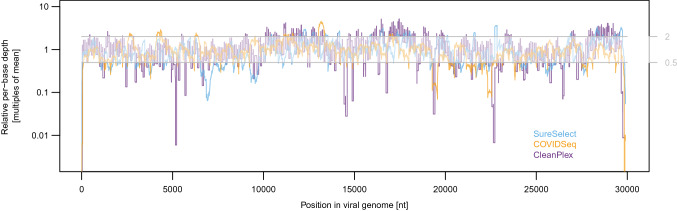
Departure from optimal depth of coverage. Relative depth was determined by comparing per-base depth to the mean depth of each library. Horizontal grey lines mark the optimal range between half the mean and twice the mean value (displayed on the right side of the y-axis).

A uniform depth of coverage across the genome would be ideal, i.e., with neither under-sequenced regions (prone to variant calling errors) nor over-sequenced regions (decreasing the depth of coverage in other regions by monopolizing the sequencing output). To quantify coverage uniformity for the three methods, we compared per-library coefficients of variation (CV) in per-base depth of coverage. COVIDSeq libraries were the most uniform (CV 54% average from all libraries), followed by SureSelect (CV 60%), then CleanPlex (CV 74%). To visualise the genomic regions with the highest dispersion, we divided the per-base depth by the mean depth of coverage for each library (Fig. 3 shows the average across all libraries). As indicated by the elevated CV, the depth of coverage is the least uniform for CleanPlex, with multiple regions systematically departing from the optimal range both below and above the mean. A histogram of per-base depth of a typical library confirming this observation is shown in Suppl. Fig. 1. The differences among regions are likely related to primer/probe design and the efficiency of their amplification/hybridization within the panel. It is noteworthy that CleanPlex kit is now available in updated version CleanPlex FLEX with degenerate primers that should address the issue of poorly performing amplicons.

### Variants

**Figure 4:**
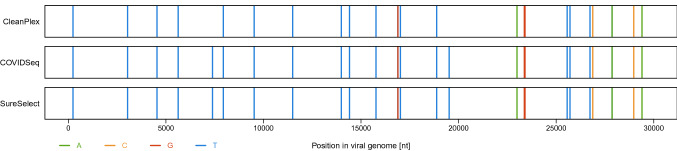
Comparison of variant calling profile of the same individual (lineage B.1.160). Vertical colored lines represent variants called; the absence of lines indicates a match with the reference sequence (accession no. NC_045512.2). Black arrows at the top show variant calls that differ among the three methods (in this case nt positions C7390T and G19518T).

In this study, 93 COVID-19 positive samples were split into aliquots and each aliquot was enriched for SARS-CoV-2 genome by a different method: CleanPlex and COVIDSeq (N = 93) and SureSelect (N = 85). Standard bioinformatic tools were used to process the raw sequencing data as described in Methods. An average library had 26 different single nucleotide polymorphisms (SNPs) compared to the reference sequence, ranging between 16 and 36 SNPs per library. Structural variants were not assessed in the present study. An example of a variant calling profile is shown in Fig. 4.

**Figure 5:**
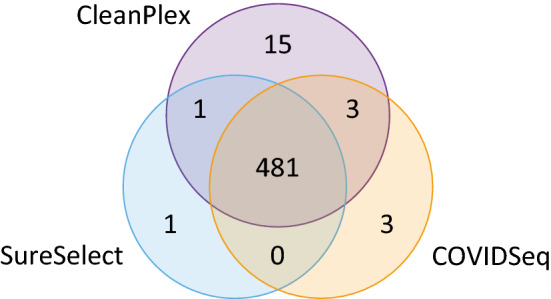
Venn diagram with the number of SNP sites called by each method. Among the 504 total SNPs sites observed in this study, 23 were not called consistently by all three methods.

SNPs called in libraries from the same individual were compared among the three methods of target enrichment. In the full set of 93 individuals, we identified 504 SNP positions, out of which 23 were not called consistently for all methods as shown in Fig. 5.

Of those, three SNPs at the 5’-end of the genome (nt positions T13C, C21T, and A27G) were only detected by SureSelect because they were outside of the panel design of both amplicon methods. Six SNPs (C5184T, T9475C, G22708T, G29734C, T29760A, and G29810T) were not correctly detected by CleanPlex due to low coverage of the particular amplicon, resulting in low SNP quality score; this variant calling error amounts to 1.2% of the total number of SNPs observed in this dataset.

Furthermore, nine SNPs were not correctly called because they were systematically located at the end of reads (CleanPlex: C5157T, C6185T, C7390T, C17999T, G19518T, T22917G, G23285T, and G29751T; COVIDseq: T19275C); this error was observed in 1.6% SNPs for CleanPlex and 0.2% for COVIDSeq. Variant calling problems with SNPs systematically located at the end of reads is likely the result of the amplicon sequencing approach, when all reads covering a particular area start and end at the same position, as is typical of CleanPlex. While COVIDSeq is also based on amplicons, its tagmentation step leads to a more even distribution of reads along the genome. The SNP that was not correctly called for COVIDSeq was adjacent to a poorly performing amplicon, thus creating an adjacent drop of coverage. For SureSelect, all reads were evenly spread along the genome regardless of probe positions, posing no particular challenge for variant calling.

The remaining five SNPs not consistently called by all three methods (A2317T, C4543T, G11083T, C26801G, and C28171T) can be attributed to variant calling error unrelated to the type of target enrichment method (likely due to their location in a region of low sequence diversity or adjacent to a deletion).

### Time, cost, scale-up and automation

All three methods include common steps of NGS library preparation with no particular technical challenges. In our experience, SureSelect requires two standard working days to build libraries in 96 reactions (rxn) format from reverse transcription to multiplex pool ready for sequencing; CleanPlex requires one and a half days; and COVIDSeq is the fastest with 1 day.

All three methods currently offer four different 96-well plates of unique dual indexes, capping the pooling capacity at 384 libraries. Thanks to its built-in normalization step, COVIDSeq libraries are easier to multiplex, resulting in a balanced pool, thus requiring less sequencing effort to achieve a required minimal coverage for all libraries. SureSelect is available in 16-rxn and 96-rxn format; CleanPlex is available in 8-rxn, 96-rxn and 384-rxn format; and COVIDSeq is available in 96-rxn and 3072-rxn format. In the 96-rxn format, the catalogue price of library preparation (not including RNA extraction or sequencing costs) is as follows: COVIDSeq 41.60 USD/library (library preparation kit with indexes, cat. no. 20049393 or 20051772); CleanPlex 60.50 USD/library (library preparation kit with magnetic beads and indexes, cat. no. 918011, 718005, and 716037); and SureSelect 127.57 USD/library (library preparation kit with indexes and Coronavirus Panel, cat. no. G9992A and 5191-6838).

At the time of writing, automated protocols are validated for the following robots (pers. comm. with technical support): SureSelect—Bravo NGS Workstation (Agilent); COVIDSeq—Bravo NGS Workstation (Agilent), Biomek i7 (Beckman Coulter), epMotion 5075t (Eppendorf), NGS STAR (Hamilton), Sciclone (PerkinElmer), Fluent DreamPrep (Tecan); and CleanPlex—Tecan and Hamilton for all steps of clean-up on magnetic beads. SureSelect and COVIDSeq kits provide sufficient reagent overage to account for dead volumes that are inevitable with liquid handling robots.

## Discussion

In this study, we compared two amplicon-based (COVIDSeq and CleanPlex) and one hybridization capture (SureSelect) methods of SARS-CoV-2 target enrichment and whole genome sequencing in high-throughput mode. All three methods yielded an excellent breadth of coverage, above 99% for all samples. SureSelect alone provided coverage in the first and last few dozens of nucleotides of the viral genome, which are not included in the primer design of either amplicon-based method. However, target enrichment with SureSelect proved to be less specific to SARS-CoV-2 and consequently, it yielded lower mapping rate and lower mean depth of coverage for SARS-CoV-2 than CleanPlex and COVIDSeq.

The depth of coverage was moderately homogeneous across the genome for COVIDSeq and SureSelect. In contrast, CleanPlex coverage was less homogeneous, yielding several systematically over- and under-represented amplicons, resulting in an occasional failure of variant calling. Coverage dropouts have already been observed for CleanPlex^32^. The problem of under-performing amplicons will likely improve with the updated primers in CleanPlex FLEX kit version. Furthermore, we observed a systematic variant calling error in both amplicon-based methods, concerning SNPs located at the first/last position of an amplicon. COVIDSeq amplicons are fewer in number and approximately twice longer than CleanPlex amplicons (see Methods for details), thus providing less scope for variant calling problems at the end of amplicons. On the other hand, having fewer amplicons would lead to a larger area without coverage in case of amplicon dropout, which can occur in highly mutated variants such as Omicron^36^. No systematic variant calling errors were detected for SureSelect in this study.

Concerning the potential for scaling up in genomic surveillance studies, multiplexing capacity is equivalent for all three kits. Fully automated protocols for different types of liquid handling robots exist for COVIDSeq and SureSelect. COVIDSeq offers a built-in normalisation step and the fastest turnaround time. Taken all together, COVIDSeq appears best suited for really high-throughput applications. However, in the wake of the worldwide crisis due to the COVID-19 pandemics, we observed that many reagents and consumables tend to be out of stock with sometimes excessive lead times, even for the most robust suppliers. In spite of their individual advantages and limitations, each of the three target enrichment kits tested in this study yielded acceptable results of whole SARS-CoV-2 genome sequencing. The independence on any one particular supplier in case of unforeseen shortage is greatly advantageous and therefore, we recommend all three kits for use in future surveillance studies.

To conclude, global genomic surveillance is essential for future management of the COVID-19 pandemics. This benchmarking study of SARS-CoV-2 sequencing methods in high-throughput context will aid as more genomic surveillance programs are called for by the WHO^30^ and particularly in countries so far under-represented in the global sequencing effort^37^.

## Materials and methods

### Ethics

This study compares different protocols using strictly de-linked and de-identified laboratory remnant samples from COVID-19 diagnostic activity, in accordance with the institutional protocol of the University of Lille, France, and in accordance with relevant guidelines and regulations. All experimental protocols were approved by the Research Ethics Committee of University Paris-Saclay. The need for informed consent was waived by the Research Ethics Committee of University Paris-Saclay. No demographic and no clinical data were recorded.

### Samples

Nasopharyngeal specimens were collected using flocked swabs and eluted in 3 mL of viral transport medium (Yocon, Beijing, China). Samples included in this study were collected between January and February 2021.

### RNA extraction

Automated nucleic acid extraction was performed using the MGIEasy Nucleic Acid Extraction Kit on the MGISP-960 instrument (BGI group, Shenzhen, China) according to the manufacturer’s instructions. The input sample volume for automated extraction was 160 µL and the elution volume was 30 µL.

### SARS-CoV-2 RT-PCR

SARS-CoV-2 detection was carried out using the TaqPath COVID-19 CE-IVD RT-PCR Kit on a QS5 thermal cycler (Thermofisher Scientific, Illkirch-Graffenstaden, France). The assay includes three viral targets (ORF, N and S regions) and an internal control (MS2 phage).

### Libraries and target enrichment

#### CleanPlex

SARS-CoV-2 Panel (Paragon Genomics, Inc., Hayward, CA, USA) was used according to the manufacturer’s instructions (version UG4001-03, Nov 2020). Reverse transcription was performed using 200 ng of RNA extracted from nasopharyngeal swabs. SARS-CoV-2 genome was amplified in two multiplex PCR (in two non-overlapping SARS-CoV-2 specific primer pools, Paragon Genomics design) with 10 cycles. Background of nonspecific PCR products was removed by digestion. Finally, unique dual indexes were introduced and each library was amplified in a PCR with 24 cycles. As per manufacturer’s instructions, a total of four purification steps was performed using CleanMag Magnetic Beads (Paragon Genomics) throughout the library preparation. Final libraries were quantified using Qubit 2.0 dsDNA HS Assay (Life Technologies). The average fragment size determined by LabChip GX system (PerkinElmer, USA) was 311 bp. Libraries were pooled in equimolar amounts. CleanPlex multiplex PCR panel contains 343 primer pairs with a median amplicon size of 149 bp, covering positions 33–29844 of SARS-CoV-2 genome.

#### SureSelect

XT HS2 RNA System (Agilent Technologies, Inc., Santa Clara, CA, USA), was used according to the manufacturer’s instructions (version A1, Sep 2020). Input of 200 ng of RNA extracted from nasopharyngeal swabs was subjected to enzymatic fragmentation followed by reverse transcription. After adaptor ligation, unique dual indexes were introduced and each library was amplified in a PCR with 14 cycles. Library quality and quantity were assessed using LabChip GX system. An input of 200 ng of indexed library was used for 90 min hybridization to SureSelect CD Pan Human Coronavirus Panel (Agilent) tenfold diluted probes as per manufacturer’s instructions. Hybridized DNA was captured using SureSelect Streptavidin Beads. The steps of capture and a series of post-capture wash steps at 70 °C were performed on liquid-handling robot Bravo NGS Workstation (Agilent) with the equivalent steps of protocol XT because it is not possible to perform a manual wash at constant temperature in 96-rxn format and protocol XT HS2 was not yet validated. Enriched libraries were amplified in PCR with 18 cycles. As per manufacturer’s instructions, a total of four purification steps was performed using SureSelect DNA AMPure XP Beads throughout the library preparation. Final libraries were quantified using Qubit dsDNA HS Assay. The average fragment size according to LabChip GX was 416 bp. Libraries were pooled in equimolar amounts. SureSelect hybridisation capture panel covers all positions of SARS-CoV-2 genome as well as of all other human coronaviruses; the panel total target size is 235 Kb.

Illumina *COVIDSeq* Test (Illumina, Inc., San Diego, CA, USA) was used according to the manufacturer’s instructions (1000000126053 v03, Feb 2021). Input of 8.5 µL of RNA extracted from nasopharyngeal swabs was subjected to reverse transcription. SARS-CoV-2 genome was amplified in two multiplex PCR (in two non-overlapping primer pools, including SARS-CoV-2 specific ARTIC v3 primers as well as several human mRNA targets for quality control purposes) with 35 cycles. The PCR product was tagmented on EBLTS HT Beads as follows. The amplicons were fragmented and tagged with adapter sequence using “Bead linked transposome” system with a built-in normalization and purification step. Tagmented amplicons were amplified in a PCR with 7 cycles. Final libraries were pooled by volume. The pool was purified using Illumina Tune Beads (which brings the total number of purification steps throughout the library preparation to two). The purified pool was quantified using Qubit dsDNA HS Assay. The fragment size of the purified pool was 384 bp, as determined in LabChip GX. COVIDSeq multiplex PCR panel contains 98 ARTIC v3 primer pairs with a median amplicon size of 392 bp, covering positions 54–29835 of the SARS-CoV-2 genome, as well as 11 primer pairs targeting human mRNA to allow the verification of correct sampling from the nasal cavity.

### Sequencing

Paired-end sequencing 2 × 150 bp was performed using NovaSeq 6000 SP Reagent Kit v1.5 (300 cycles) according to the manufacturer’s instructions. All three runs passed our usual quality control steps at run level, such as clusters passing filter: 73–85% and phred quality score above 30%: 92–93%. Raw sequencing data passed our usual quality checks using fastp v0.20.1, fastqc v0.11.9, and multiqc v1.9 software. In order to compare data generated with equivalent sequencing effort for each method, we downsampled the FASTQ files to 2 M read pairs per library with seqtk v1.3.106 using seqtk-sample command^38^.

### Bioinformatics

Each of the three kits requires specific bioinformatic treatment due to their different design. For SureSelect, unique molecular identifier (UMI) sequences were extracted and adaptor sequences removed with Agilent Genomics NextGen Toolkit v2.0.5 (AGeNT)^39^ using AGeNT-trim command, keeping reads with minimum read length of 50% of the original read length after trimming. Reads were aligned to the reference genome (NC_045512.2) using bwa-mem2 v2.2.1^40^ for all three methods.

For SureSelect, read pairs with UMI information were tagged and duplicates were merged using AGeNT-locatit command with default parameters. For CleanPlex and COVIDSeq, the primer sequences were hard-clipped with SAMtools v1.11^41^ using samtools-ampliconclip command, using strand information from bed file, clipping on both ends and marking as failed reads < 30 bases.

For all three methods, the depth of coverage was examined using samtools-mpileup, disabling per-Base Alignment Quality, keeping anomalous read pairs, skipping reads with mapQ < 1 and skipping bases with quality < 1, excluding flags UNMAP,SECONDARY,QCFAIL. The mapping rate was assessed using samtools-flagstat command. Variants were called using Octopus v0.7.4^42^ in very fast mode with polyclone calling model (--very-fast --min-phase-score 30 --organism-ploidy 1 --downsample-above 8000 --downsample-target 5000 --allow-octopus-duplicates --good-base-quality 30 --min-good-bases 30 --min-mapping-quality 40 -C polyclone --min-clone-frequency 0.01 --max-clones 4). VCF was normalized with BCFtools v1.11 using bcftools-norm command^43^, splitting multi-allelic sites. Normalised VCF was filtered using bcftools-filter command, keeping variants with QUAL > 2000, MQ > 40, and AF > 0.50. Statistical analyses were performed and figures were generated in R^44^ with basic functions and the library “vioplot”^45^.

To assign samples to specific lineages, we used BCFtools-consensus command to generate a fasta file from the filtered VCF. Lineage assignment was performed using Pangolin v2.3.8^46^, with pangoLEARN database version 21/04/2021 and with maximum 10% of Ns allowed.

## Supplementary Information

Supplementary Figure 1.

## Data Availability

The data generated and analysed in this study are available in the European Nucleotide Archive (EMBL-EBI) under accession number PRJEB52218 (http://www.ebi.ac.uk/ena/data/view/PRJEB52218).
