# Supplementary material for: A comparison of high-throughput SARS-CoV-2 sequencing methods from nasopharyngeal samples
Source: Sci Rep. 2022 Jul 22;12:12561. doi: 10.1038/s41598-022-16549-w (PMC9306416; doi:10.1038/s41598-022-16549-w)
Supplement: Supplementary file 1 — Supplementary Figure 1. [file 41598_2022_16549_MOESM1_ESM.pdf]

A comparison of high-throughput SARS-CoV-2 sequencing methods from nasopharyngeal samples

Zuzana Gerber, Christian Daviaud, Damien Delafoy, Florian Sandron, Enagnon Kazali Alidjinou, Jonathan Mercier, Sylvain Gerber, Vincent Meyer, Anne Boland, Laurence Bocket, Robert Olaso, Jean-François Deleuze.

Supplementary Figures

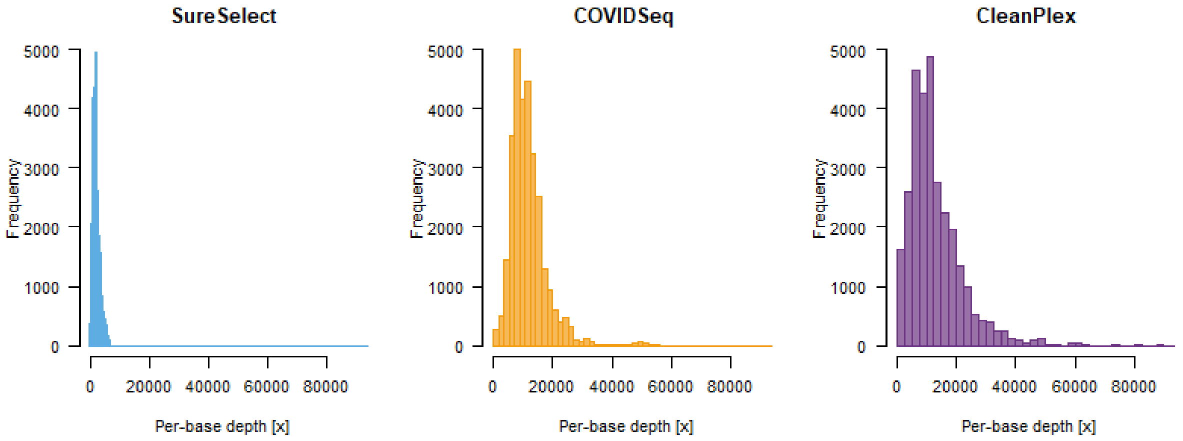

Suppl. Figure 1. ***A histogram of per-base depth of a typical library.*** *A wider distribution confirms heterogeneous coverage for CleanPlex. Furthermore, higher frequency in the leftmost bin indicates the presence of poorly covered regions in case of CleanPlex.*
